# Imagining the way forward: A review of contemporary motor imagery theory

**DOI:** 10.3389/fnhum.2022.1033493

**Published:** 2022-12-22

**Authors:** Austin J. Hurst, Shaun G. Boe

**Affiliations:** ^1^Laboratory for Brain Recovery and Function, School of Physiotherapy, Dalhousie University, Halifax, NS, Canada; ^2^Faculty of Health, Dalhousie University, Halifax, NS, Canada; ^3^Department of Psychology and Neuroscience, Dalhousie University, Halifax, NS, Canada; ^4^School of Physiotherapy, Dalhousie University, Halifax, NS, Canada; ^5^School of Health and Human Performance, Dalhousie University, Halifax, NS, Canada

**Keywords:** motor imagery, action planning, motor learning, functional equivalence, mental imagery, mental practice

## Abstract

Over the past few decades, researchers have become interested in the mechanisms behind motor imagery (i.e., the mental rehearsal of action). During this time several theories of motor imagery have been proposed, offering diverging accounts of the processes responsible for motor imagery and its neural overlap with movement. In this review, we summarize the core claims of five contemporary theories of motor imagery: motor simulation theory, motor emulation theory, the motor-cognitive model, the perceptual-cognitive model, and the effects imagery model. Afterwards, we identify the key testable differences between them as well as their various points of overlap. Finally, we discuss potential future directions for theories of motor imagery.

## 1. Introduction

Motor imagery, defined here as the mental rehearsal of action without engaging in actual movement (Moran et al., 2012), has been a subject of interest across a number of fields. This interest stems in large part from evidence showing motor imagery can drive acquisition of simple and complex motor skills (Schuster et al., 2011; Ladda et al., 2021). For example, sport psychologists have found that mental practice can be an effective supplement to physical training for athletes (Weinberg, 2008; Collet et al., 2011). Similarly, practicing surgical techniques via motor imagery has been associated with increased surgical skill (Sevdalis et al., 2013; Cocks et al., 2014). For motor recovery during stroke rehabilitation, there is a growing body of evidence suggesting that imagery-based interventions can be an effective supplement to physical therapy (Dickstein and Deutsch, 2007; Barclay et al., 2020). Despite these promising results, the effects of imagery training are highly variable across studies along with equally-inconsistent methods (Ladda et al., 2021), highlighting the need for more research (see Gaughan and Boe, 2022 for a review of variability in stroke-related motor imagery interventions).

To advance both basic and applied research on motor imagery, it is important to understand how it works: its neural correlates, its cognitive demands, and the processes that give rise to the experience of mentally simulating movement. However, despite decades of research, the exact mechanisms behind motor imagery have remained poorly understood. Over the past two decades many theories of motor imagery have been proposed, attempting to explain different aspects of the process. Although these theories have many similarities, they also make conflicting claims on key issues with no clear consensus as to which theory elements are correct. Therefore, to improve our understanding of the mechanics of motor imagery, it is important to review the existing theories, compare their claims, and identify their key testable disagreements.

Here we review five modern theories of motor imagery: the motor simulation theory (MST), the motor emulation theory (MET), the motor-cognitive model (MCM), the perceptual-cognitive model (PCM), and the effects imagery model (EIM)^1^ (for the purpose of this review, the only difference between a “theory” and a “model” is how they were named by their authors). First, we introduce and briefly summarize each model. Then, we examine how the claims of these theories differ on key questions, such as how they believe imagery mechanistically differs from physical execution, and how learning through imagery practice is possible. Finally, we identify the most theoretically interesting differences in prediction between models and discuss future directions for motor imagery theory. Note that an evaluation of the evidence for each theory's claims is outside the scope of this review: instead, our aim was to summarize current theory in the field and discuss the key similarities and differences between these theories.

**Figure 1:**
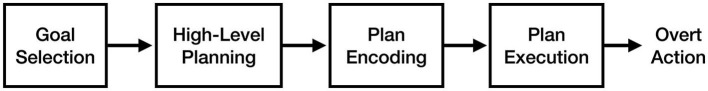
The key stages of the motor execution process, based on Jeannerod (1995).

To make it easier to identify differences and similarities between theories, this review defines them relative to four key stages of motor execution: goal selection, high-level planning, plan encoding, and plan execution (see Figure 1). Goal selection is the stage of action where the current motoric goal (e.g., picking up a mug) is selected. The high-level planning stage involves the translation of a goal into a high-level motor plan to achieve it (e.g., reaching out and wrapping your fingers around the mug handle). The plan encoding stage, also referred to as 'motor programming', involves translating the high-level motor plan into muscle-specific motor signals (e.g., “increase tension in the right anterior deltoid by 20%”). Finally, the plan execution phase consists of sending the encoded motor plan to the muscles to produce overt action. These stages are based on those proposed by Jeannerod (1995), albeit with different names. Processes that are thought to occur during action but not imagery (e.g., overt action) are shown on subsequent figures in light gray.

## 2. Theory summaries

### 2.1. Motor simulation theory

Proposed formally in Jeannerod (2001) and expanded upon in later work (Jeannerod, 2004, 2006; see O'Shea and Moran, 2017 for a review), motor simulation theory (MST) claims that motor imagery and motor execution use the same neural processes up to the point of muscle activation and resulting movement, which is suppressed by an inhibitory mechanism at some point between plan encoding and overt action. In other words, MST asserts that performing an action and performing imagery of an action are *functionally equivalent*, with motor imagery being the experience of *covert action* (the internal stages of motor execution) without *overt action* (i.e., physical movement). Since its introduction MST has been widely popular, with Jeannerod (2001) having over 1,600 citations (Scopus, 2022).

**Figure 2:**
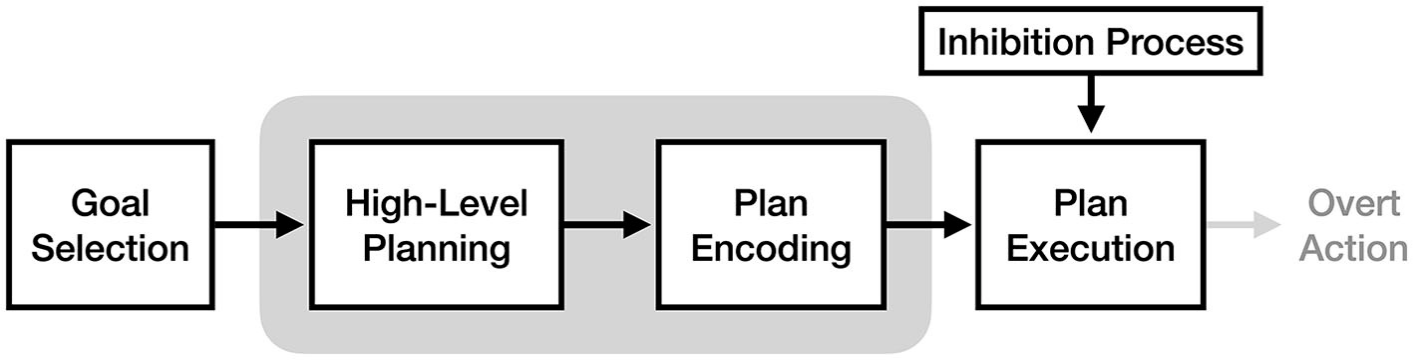
The process of motor imagery as proposed by motor simulation theory. The stages of motor execution thought to be responsible for the experience of motor imagery are enclosed in gray.

As illustrated in Figure 2, motor simulation theory claims that the experience of motor imagery is a product of the high-level planning and/or plan encoding processes, and that these processes are identical between motor imagery and motor execution. It does not make clear assertions about the exact process responsible for imagery, only that it involves the motor cortex and its descending motor pathways (in addition to pre-motor regions involved in action), and that covert action resulting from motor imagery would be in a “true *motor* format” (Jeannerod, 2001). This lack of mechanistic detail, as discussed in O'Shea and Moran (2017), makes MST both flexible and difficult to falsify.

A key component of motor simulation theory is its inhibitory mechanism, which prevents the motor commands generated during imagery from causing actual movement. Jeannerod (2001) proposes two possible ways this mechanism might be implemented: 1) a process inhibiting motor commands from descending past the brainstem or spinal cord, or 2) a reduced level of motor cortex activation during imagery, resulting in output below the threshold required to activate motor neurons in the spinal cord (and thus below the threshold required for movement). Regardless of its implementation^2^, MST's core claim of functional equivalence requires this inhibitory process to exist: without it, it would not be possible for motor imagery to make use of the neural motor pathways without producing movement.

Jeannerod (2001) offers two main areas of evidence in support of MST. The first is similarity in mental chronometry; that generally, imagery of an action takes about as long as performing the action, with physical and ergonomic constraints affecting motor imagery and physical movement to a similar degree (e.g., slower reaction times in a tapping task when targets are further away, Sirigu et al., 1995). Jeannerod (2001)'s second line of evidence for MST is the similarity in neural activation between motor imagery and actual movement, reviewing 15 fMRI studies and finding that both imagined and overt action increased activation in the central and cingular gyri, supplementary motor area (SMA), and inferior parietal lobule (IPL).

### 2.2. Motor emulation theory

**Figure 3:**
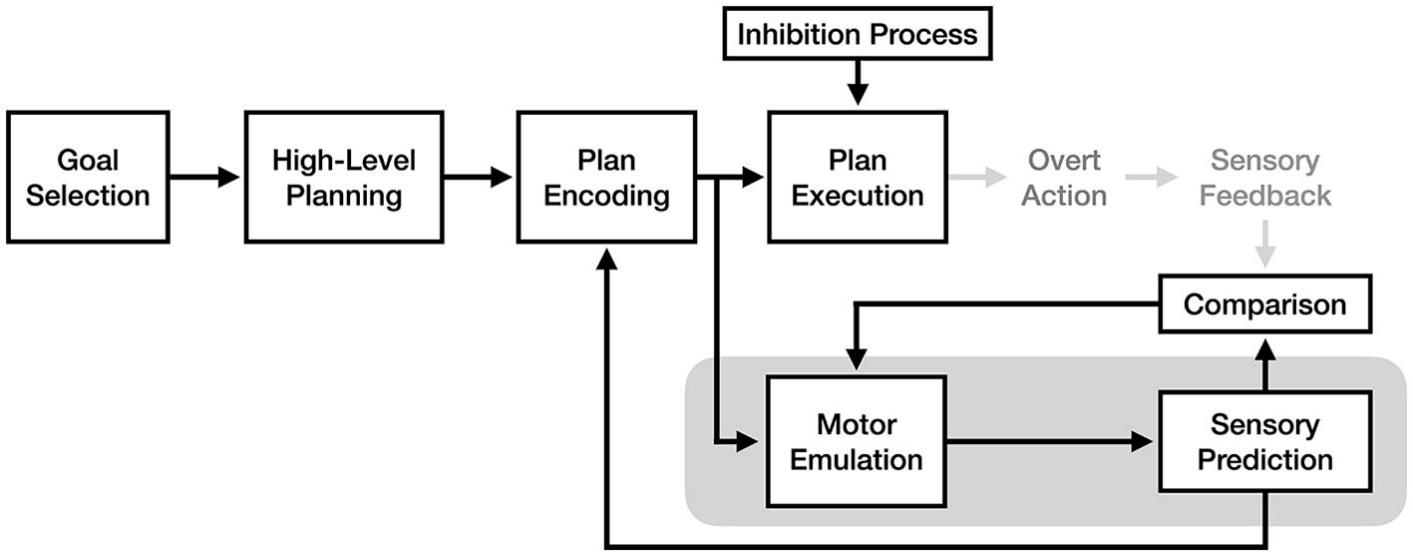
The process of motor imagery as proposed by motor emulation theory. The stages of motor execution thought to be responsible for the experience of motor imagery are enclosed in gray.

Drawing on concepts from the engineering fields of signal processing and control theory, motor *emulation* theory (MET) proposes a detailed model of how the motor system adapts and learns using sensory input (Grush, 2004). Importantly, this theory of motor control also makes unique and specific claims about how and why motor imagery is produced within the motor system. The core claim of MET is that the motor system involves an *emulation* process, which takes a copy of an encoded motor plan as input and predicts its sensory consequences (a process described in other work as creating a “forward model” of a motor plan^3^, see Wolpert et al., 1995). According to the theory, the purpose of the emulator is to provide fast feedback to the plan encoder during movement, allowing for corrections in direction or force in an action to be made without relying on higher-latency sensory input. The experience of motor imagery is thus the experience of the emulator's predictions when motor execution is inhibited (see Figure 3).

An important claim of motor emulation theory is that the motor emulator is adaptive and improves the accuracy of its predictions over time using a feedback mechanism. Specifically, the theory proposes that the emulator's sensory predictions are actively compared to the actual sensory results of action during motor execution, and that the differences between prediction and outcome are continuously used to adjust the emulator's future predictions. Although prior work on forward models had already proposed the concept that motor learning was based on an active comparison process with sensory predictions (Jordan and Rumelhart, 1992; Miall and Wolpert, 1996), the novel contribution of MET is that the forward modeling process and the motor imagery process are one and the same, with imagery being the conscious experience of a forward model's sensory predictions.

One of the more specific proposals of Grush (2004) is that the emulation mechanism follows the same principles as a Kalman filter (a statistical process commonly used for visual and motor processing in robotics). In addition to the basic adaptive emulation process described above, a Kalman filter system would also estimate the “noisiness” (i.e., accuracy) of sensory input during movement and adjust the amount of Kalman gain (correction applied to the emulator) accordingly. During motor imagery the Kalman gain would be zero, meaning that mental practice (defined here as motor imagery done with the intent to improve performance) would presumably not change the emulator's predictions. Additionally, a Kalman-based emulator would involve what Grush (2004) describes as an *articulated model*: a set of state variables representing the full musculoskeletal system and its corresponding sensory projections. An emulator implemented in this way would a) be able to keep a full representation of the state of the body and its muscles at all times, and b) would be able to simulate encoded motor plans in their true motoric form (i.e., their effects on specific muscles).

However, Grush (2004) is careful to note that the above Kalman-based emulator is only one possible implementation of a motor emulator, and that other implementations (such as an associative mechanism relating motor commands to their prior sensory results) are possible. As such, it may be useful to consider MET as having two different forms: *core* emulation theory, which is implementation-agnostic, and *Kalman* emulation theory, which makes specific claims about how the emulator is implemented.

In support of motor emulation theory, Grush (2004) points to prior evidence and theory in favor of a sensory prediction and feedback mechanism in the motor system (e.g., Wolpert et al., 1995). Regarding MET's account of motor imagery, Grush (2004) cites much of the same behavioral and neural evidence for functional equivalence as Jeannerod (2001) does in favor of motor simulation theory but argues that MET provides a clear and specific mechanism for the sensory experience of motor imagery while MST does not.

### 2.3. Motor-cognitive model

Proposed by Glover and Baran (2017), the motor-cognitive model (MCM) is a recent theory of motor imagery that rejects the idea of “functional equivalence” proposed by the motor simulation theory. In the MCM, motor imagery and execution are thought to use the same mechanisms for movement planning but diverge at the point of execution, with the generation of imagery having different cognitive demands than performing and monitoring physical movement. Specifically, in the absence of external sensory input from a movement (which can be monitored passively), motor imagery is thought to use different cognitive pathways to actively create, elaborate, and monitor an internally-generated multi-sensory image.

**Figure 4:**
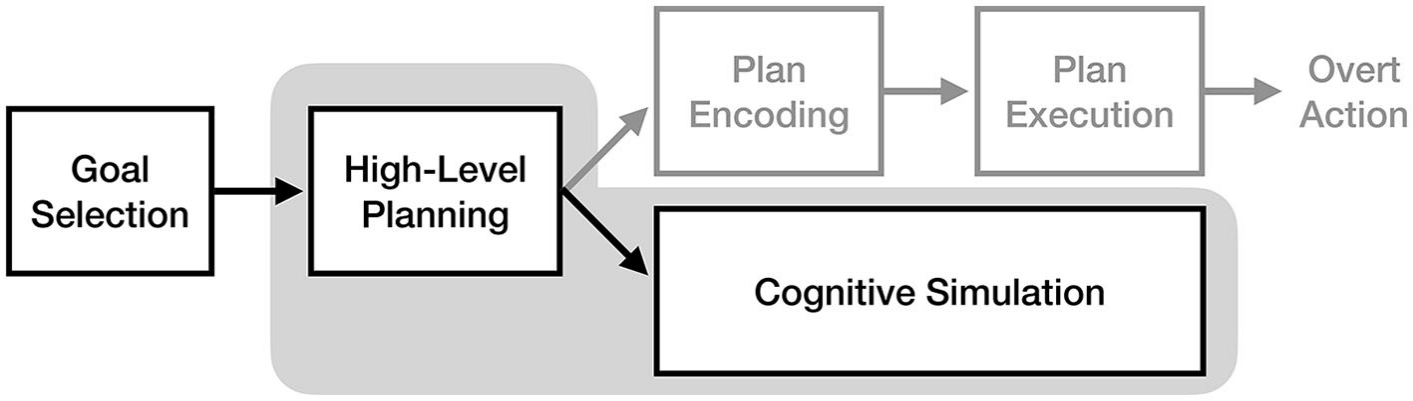
The process of motor imagery as proposed by the motor-cognitive model. The stages of motor execution thought to be responsible for the experience of motor imagery are enclosed in gray.

As illustrated in Figure 4, the motor-cognitive model asserts that motor imagery and motor execution only rely on the same neural pathways up to the point of movement planning, after which they diverge. Glover and Baran (2017) describe the “initial” motor image as being generated by the planning process, which is then consciously elaborated through cognitive simulation. This claim has two important implications: first, as noted by Glover and Baran (2017), imagery should be more cognitively demanding for novel actions than well-practiced actions, as more cognitive effort will be needed to create and elaborate a poorly-defined initial image. Second, the direct pathway from planning to cognitive simulation implies that motor plans are never converted into a true “motor command” format during the imagery process.

Given that imagery is thought to rely more on executive and attentional resources than action, a key prediction of the motor-cognitive model is that tasks that place demands on the same resources used for imagery should impair mental practice to a greater extent than physical practice. Additionally, given the increased need for “elaboration” for poorly-represented actions, dual-task interference should impair imagery for novel actions to a greater extent than well-practiced actions. However, the MCM is not explicit as to which cognitive resources are thought to be involved in “elaboration” during imagery, making specific predictions about interference from specific cognitive tasks difficult.

An additional difference in the cognitive demands of imagery and action highlighted by Glover and Baran (2017) is that imagery tasks generally require context switching. For example, an experimental task might ask participants to press a button to indicate they have completed a mental action, meaning that focus must be shifted between the imagined action and response action. However, this cognitive difference is largely limited to experimental contexts.

In support of the motor cognitive model, Glover and Baran (2017) cite evidence at odds with the motor simulation theory's core claim of 'functional equivalence'. Specifically, the authors highlight that although the mental chronometry of motor imagery and overt action is similar for simple and previously-rehearsed movements, their differences in timing increase for both complicated and novel actions that require more cognitive processing (e.g., Calmels et al., 2006). Additionally, Glover and Baran (2017) found that occupying cognitive resources with backwards counting during a reaching task impaired movement times for motor imagery considerably more than for overt action, a pattern they argue is consistent with the MCM but not the MST. Moreover, the authors point to various neural dissociations between motor imagery and overt action, such as motor imagery involving more activation in frontal areas related to executive processing (Guillot et al., 2009) and dissociations between motor imagery and action performance in patients with brain injuries (McInnes et al., 2016), both of which are presented as evidence against the MST's claim of functional equivalence.

### 2.4. Perceptual-cognitive model

The perceptual-cognitive model (PCM), as described in Frank and Schack (2017), proposes that motor imagery is the conscious experience of high-level motor planning. Specifically, this theory argues that motor plans for simple actions (e.g., bending an elbow) and their sensory results (e.g., the look and feel of bending an elbow) are closely associated and stored together neurally as units, such that the selection of a motor plan will likewise activate its associated sensations. These motor/sensory units, referred to as Basic Action Components (BACs) by the model, are thought to be the building blocks from which all complex motor plans are created.

**Figure 5:**
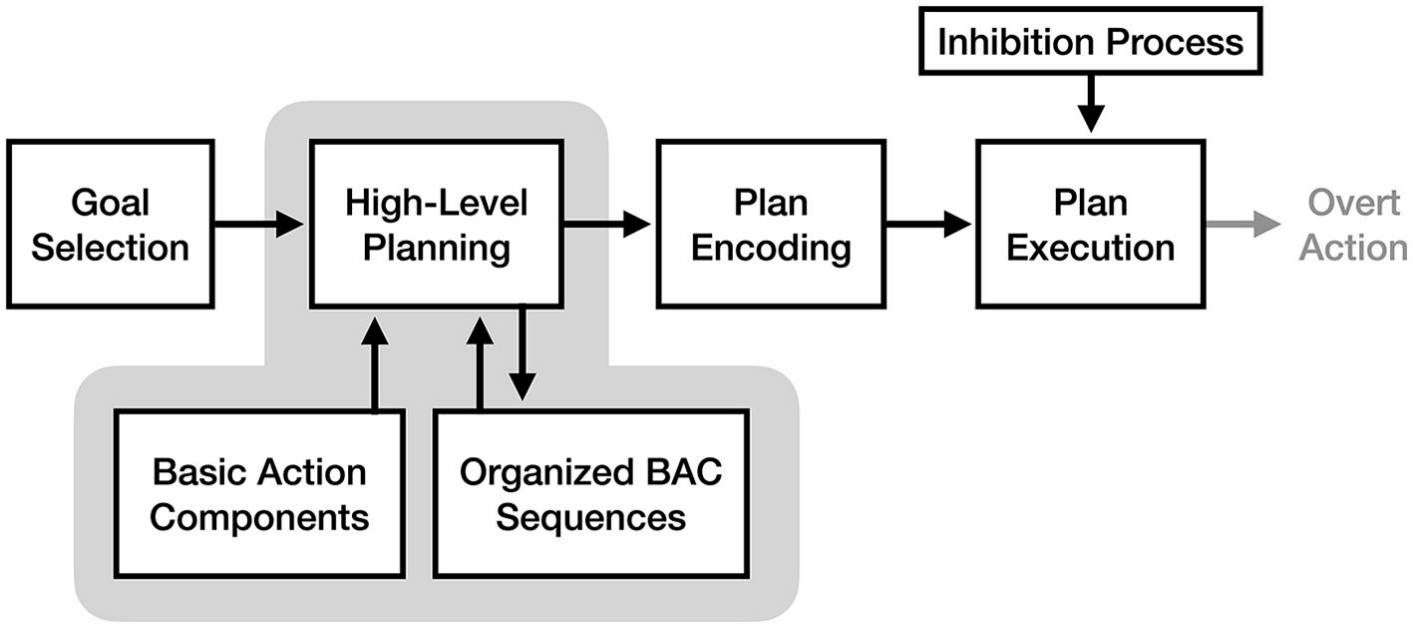
The process of motor imagery as proposed by the perceptual-cognitive model. The stages of motor execution thought to be responsible for the experience of motor imagery are enclosed in gray.

According to the perceptual-cognitive model, a key function of high-level motor planning is the cognitive restructuring of BACs for easier future retrieval. Frank and Schack (2017) refer to this as “perceptual-cognitive reorganization” and “scaffolding,” a process where common patterns of BACs are grouped into organized sequences. These sequences can then be retrieved as a whole during future planning, reducing the cognitive demands of future planning (see Figure 5). As such, the PCM views motor imagery as the conscious experience of retrieving and organizing BACs into a motor plan in the absence of any overt action. An important implication of this theory is that because motor imagery is focused exclusively on the high-level planning process, imagery should result in faster improvements to BAC organization than equivalent physical practice (where attention is split between motor planning and sensory feedback). This is notable because despite agreeing with the idea of “functional equivalence” between the cognitive processes used for motor imagery and action, the model predicts differences in the allocation of attention to those processes between imagery and action will have diverging effects on motor learning.

In support of the perceptual-cognitive model, Frank and Schack (2017) cite prior evidence supporting the idea that cognitive representations of actions are hierarchically structured. Specifically, they note that experts in a given task have more structured action representations for that task than novices (e.g., conceptualize a tennis swing as three distinct phases of movement rather than a single motion, Schack and Mechsner, 2006); and that practicing a complex action increases the degree to which its representation is consciously structured and grouped (Frank et al., 2013). As evidence for the PCM's claim that motor imagery is the experience of rehearsing and restructuring action components, Frank and Schack (2017) assert that while physical practice of an action results in better performance than imagery practice, motor imagery results in more elaborate and expert-like structured representations of the movement (Frank et al., 2014).

### 2.5. Effects imagery model

Recently proposed by Bach et al. (2021), the effects imagery model (EIM) flips traditional thinking regarding motor imagery on its head: whereas prior theories view motor imagery as occurring after or during the motor planning process, the EIM argues that imagery is also an essential part of the goal selection process *prior* to motor planning. Unlike traditional concepts of motor imagery, which involve mentally simulating the *experience* of a motor plan, this form of imagery (termed “effect imagery” by the authors) involves bringing to mind the desired *effects* of the action in order to activate a relevant motor plan via association. This proposal draws heavily from ideomotor theories of action initiation, which are likewise based on the idea that actions are caused by activation of their mental representations (see Shin et al., 2010 for a review).

**Figure 6:**
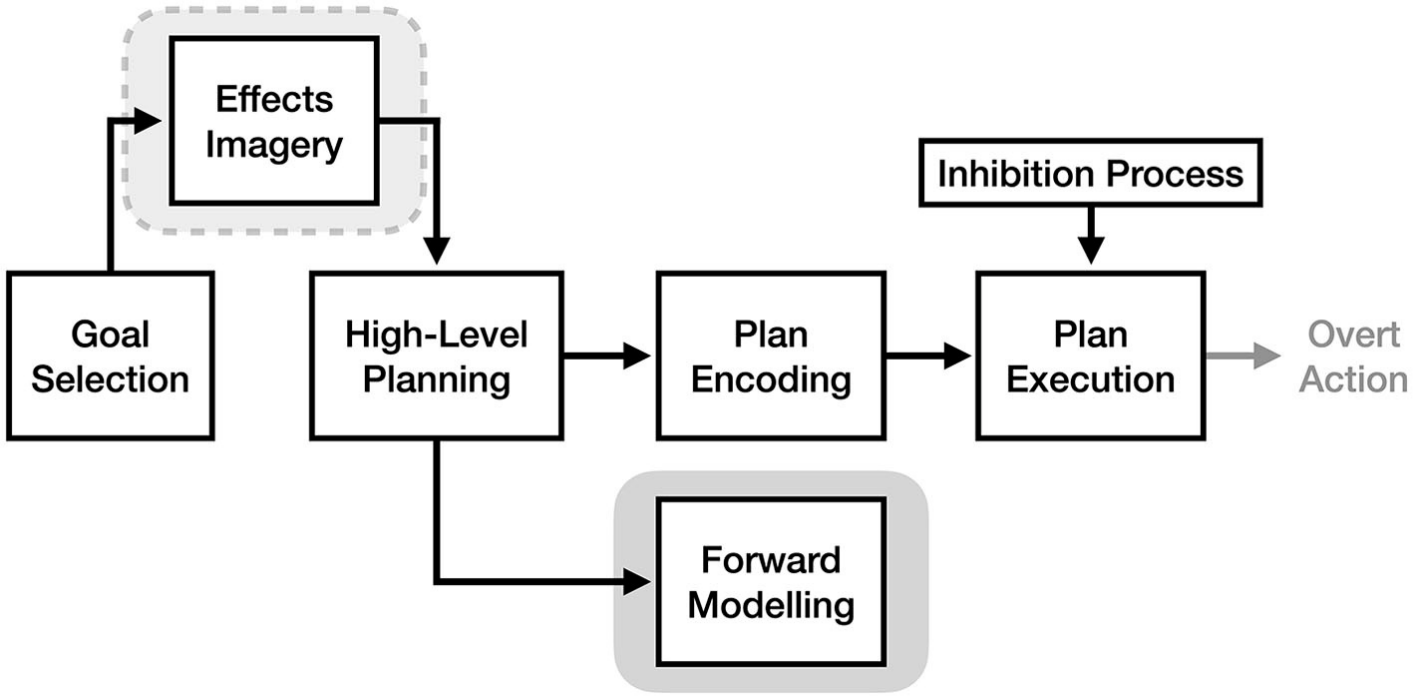
The process of motor imagery as proposed by the effects imagery model. The stage of motor execution thought to be responsible for the experience of effects imagery is enclosed in light gray. The stage responsible for traditional motor imagery is enclosed in dark gray.

As illustrated in Figure 6, the effects imagery model asserts that imagery of the desired effects of an action is necessary for initiating high-level motor planning based on an initial goal. According to the theory, effects imagery bridges goals and motor plans because of the strong bidirectional associations between mental representations of actions and their sensory effects, such that bringing to mind the effects of an action will in turn activate a related motor plan. For example, the EIM argues that to pick up a cup, you first bring to mind the image of holding the cup, which activates a basic motor plan of how to accomplish that goal based on prior experience. Importantly, effects imagery is not required to be detailed or even fully conscious (e.g., a stray memory of playing catch as a child reflexively brings your hand into a ball-holding shape): as long as the image is sufficient to prime a relevant motor plan, it will be able to initiate action.

Although the effects imagery model argues that *most* imagery in the motor system is imagery of effects, the framework also explicitly allows for the vivid simulation of generated motor plans (i.e., traditional motor imagery) to optionally take place. Bach et al. (2021) views this as a separate form of motor imagery that we can optionally use for the purpose of practicing an action mentally. In other words, the EIM proposes that there are two forms of motor imagery: a brief and reflexive form (effects imagery) that *initiates* motor planning, and a detailed, effortful form (traditional motor imagery) that optionally *follows* motor planning (see Figure 6). This second form of motor imagery is thought to be needed when trying to extract information not present in its basic effects image (e.g., how long a movement will take, the position of a limb at a given point in time, the spatial constraints involved in a movement, etc.). The EIM suggests that traditional motor imagery is the conscious experience of an associative process, whereby bringing to mind a given motor plan via effects imagery likewise activates *additional* sensory predictions associated with those actions. Relatedly, although the EIM implies that high-level motor planning activates downstream plan encoding processes to some extent (as it claims an inhibitory mechanism is required to prevent movement), the experience of motor imagery does not depend on plan encoding in this model.

Bach et al. (2021) offer a range of neural and behavioral findings in support of the effects imagery model. Regarding the idea that actions are initiated by effects imagery, they highlight evidence showing close association between neural representations of actions and their effects (e.g., neurons in the pre-motor cortex that respond equally to actions and their perceived auditory/visual results, Kohler et al., 2002). Additionally, they note that people are more likely to accidentally perform an action they were asked to only imagine when presented with a visual cue of the action's effects, and that this only occurs when the cued effects are consistent with the imagined action (Colton et al., 2018). In support of the EIM's suggestion that plan encoding is not essential to motor imagery, Bach et al. (2021) cite evidence showing neural overlap between motor imagery and action is more reliable in movement planning structures (e.g., pre-motor cortex) than movement execution structures (e.g., primary motor cortex). Notably, they cite an fMRI study where phantom limb patients were asked to imagine moving or to 'actually move' their amputated limb: in both cases no actual movement or sensory feedback was produced, but notable differences in motor cortex and cerebellar activity were still observed between the motor imagery and 'action' conditions (Raffin et al., 2012).

## 3. Comparing theories of motor imagery

### 3.1. Where does motor imagery come from?

Given that we can consciously experience the act of motor imagery, an obvious question for any theory of motor imagery is “where does that conscious experience come from in the proposed model?.” On this matter, the theories under review can be broadly split into two groups: theories that argue imagery occurs before plan encoding, and theories that claim it occurs afterwards.

In the former category (pre-encoding models), the motor-cognitive model (MCM), perceptual-cognitive model (PCM), and the effects imagery model (EIM) all argue that the experience of motor imagery arises from the high-level planning stage of the motor process, either as part of the planning process itself (PCM), a subsequent elaboration process (MCM), or the sensory associations of a given plan (EIM). In the latter group (post-encoding models), the motor emulation theory (MET) claims that the experience of motor imagery arises after plan encoding as a consequence of emulating the sensory consequences of encoded motor plans. Motor simulation theory (MST) is not specific enough about the origins of conscious imagery to be categorized as either pre-encoding or post-encoding but could be interpreted either way: as a functional equivalence model, any conscious components of motor planning during normal movement might likewise be responsible for imagery. On the other hand, Jeannerod (2001) suggests that the activation of the descending motor pathways during imagery could contribute to “corollary signals that propagate upstream” and references a paper on forward modeling (Wolpert et al., 1995), highlighting the possibility that imagery in MST could result from post-encoding processes.

The key distinction between pre-encoding imagery theories (MCM, PCM, EIM) and post-encoding imagery theories (MCT, potentially MST) is that post-encoding theories require that motor plans are encoded into a true motor form (e.g., “relax left tricep 20%”) before imagery can occur. This distinction has several important consequences: first, that the motor system must have a mechanism for converting encoded motor plans back into a consciously-accessible sensory format (e.g., the emulator proposed by MET). Second, that any process distorting or otherwise interfering with the encoding of motor plans should likewise interfere with the accuracy of motor imagery. Third, that the brain regions associated with motor plan encoding should be active during imagery (there is evidence in support of this, see Hétu et al., 2013; Hardwick et al., 2018). On this last point, it is important to note that activity in planning regions is not incompatible with pre-encoding imagery theories: although plan encoding is not essential to the imagery process in these models, the plan encoding regions may still be activated as a result of activation in the high-level planning regions immediately upstream.

In addition to the above groups of thought, the effects imagery model also argues that a specific form of motor imagery (“effects imagery”) *initiates* high-level motor planning and thus occurs just prior, acting as an associative bridge between goal selection and high-level planning. However, this form of imagery is distinct from “motor imagery” as traditionally defined (i.e., imagery of *performing* an action), and should thus not be compared directly to other theories.

### 3.2. How is learning through motor imagery possible?

To understand how motor imagery works, it is important to account for how mental practice, in the absence of any external feedback, can improve motor performance on a range of tasks. Out of the theories under review, there are two broad groups of proposed learning mechanisms: long-term potentiation, in which the mental simulation of action strengthens the neural connections involved in those movements through repeated activation, and plan improvement, in which the mental rehearsal of an action allows for its high-level motor plan to be improved.

The theory that most emphasizes learning via long-term potentiation is the motor simulation theory, which argues that since imagery uses the same neural pathways as actual movement, mental practice should facilitate future execution of that by reinforcing the pathways involved in selecting and encoding it (Jeannerod, 2001). This theory thus predicts that all components of the motor system involved in an action, up to the point of execution, should be similarly reinforced through motor imagery. Relatedly, the effects imagery model argues that motor imagery promotes learning by strengthening the bi-directional associations between motor plans and their perceptual effects via long-term potentiation, thus making it quicker to retrieve the motor plan required to achieve a desired effect and likewise strengthening the sensory predictions associated with given motor plan (Bach et al., 2021). In contrast to MST, the EIM argues that the benefits of mental practice are focused on high-level cognitive associations as opposed to being generalized throughout the downstream motor system.

Of the theories arguing that motor imagery improves motor planning, there are two main mechanisms they propose for the source of this improvement: the reorganization of motor plans, and the simulation of motor plans. The perceptual-cognitive model argues for the former, claiming that the process of imagery helps break complex movements into simple organized chunks, making them easier to retrieve in that sequence in future. By contrast, the motor emulation theory and motor-cognitive model both suggest that motor imagery improves performance by simulating the effects of motor plans, thus providing feedback that can be used to improve subsequent plans. In the MET this is achieved through the emulation mechanism, which simulates the sensory consequences of motor plans and feeds them back to the high-level motor planning stage, allowing for useful re-evaluation or fine-tuning of the original motor plan. An important implication of this is that the benefits of mental practice should depend strongly on the accuracy of the emulator's predictions: if trained with inaccurate sensory feedback, motor imagery training would presumably *impair* motor learning by reinforcing incorrect high-level plans and motor-sensory emulator mappings. The motor-cognitive model likewise implies that imagery facilitates learning through simulation, but through a different mechanism: instead of emulating encoded motor plans, the MCM simulates the effects of a plan by taking its “initial motor image,” elaborating its details, and actively monitoring the progress of the imagined movement. Although the MCM's authors do not provide an account of how motor imagery facilitates learning in their model (Glover and Baran, 2017), the MCM's simulation of motor plans provides an obvious potential mechanism: if the motor image is sufficiently detailed, it should be able to provide useful feedback for future motor planning. Differences in prediction between the MCM and MET's simulation mechanisms include that the MCM's mechanism should depend more on the fidelity of the visual imagery involved in a motor image, and that unlike the MET, the MCM's simulations should have no effects on the motor plan encoding process.

The question of whether learning occurs through simulated feedback or cognitive reorganization is important, as they make differing predictions about when motor imagery practice is most effective. If the PCM is correct, training that focuses on improving the cognitive representations of basic motor components and facilitates the chunking process should be highly effective. If the MET is correct, successful imagery training should depend greatly on the accuracy of the emulator's predictions and should allow improvements to plan encoding as well as high-level planning. However, both theories predict that motor imagery practice requires basic prior experience with the components of the imagined movement in order to be effective, as such experience is necessary to have perceptual-cognitive representations to organize (PCM) or sufficiently-accurate emulator mappings to generate predictions (MET).

It is important to note that long-term potentiation and motor plan improvement are not mutually-exclusive methods of learning-via-imagery. For instance, while the motor emulation theory focuses on learning through plan improvement, it also involves the mental rehearsal of the motor encoding process which would presumably facilitate future encoding of the same plans. Likewise, the effects imagery model argues that in addition to the strengthening of perceptual-motor associations, the reorganization of motor plans (as proposed in the PCM) may also contribute to the benefits of mental practice. Similarly, the reorganization and simulation mechanisms for motor plan improvement are entirely compatible: despite being proposed separately in different theories, motor imagery could also facilitate learning through both methods concurrently.

### 3.3. What modalities are involved in motor imagery?

Although all theories under review discuss the experience of “motor imagery,” they make differing claims about what sensory modalities (e.g., visual, auditory, kinaesthetic/proprioceptive) are involved in that experience. Some theories argue that motor imagery is predominantly *unimodal*, involving kinaesthetic imagery (i.e., how it would feel for the muscles and body to perform a given action). Other theories are explicitly *multimodal*, arguing that imagery of performing a movement involves experiencing how it would look and/or sound to perform an action in addition to kinaesthetic imagery.

The motor-cognitive model, perceptual-cognitive model, and effects imagery model are best categorized as multi-modal theories of imagery. In the MCM, imagery is a visual and kinaesthetic mental simulation of how it would look and feel to perform an action, drawing on experience to create and actively monitor a spatial image of the body performing the movement. Although the MCM does not explicitly reference auditory imagery, the “elaboration” process based on generalized cognitive networks presumably allows for imagery from any modality. Similarly, the PCM argues that our cognitive representations of basic actions are tightly associated with our representations of their sensory consequences, which would presumably involve multiple modalities. The EIM argues explicitly that motor imagery is multi-modal, involving the experience of visual, auditory, and proprioceptive elements.

In contrast, the motor emulation theory is best categorized as a unimodal theory of imagery. As presented in Grush (2004), MET's emulation mechanism involves a sensory feedback loop: encoded motor plans are converted into sensory predictions, which are then compared to the plans' actual sensory results to improve the emulator's future predictions. Because the emulator is thought to function by simulating the states of all the joints, ligaments, and proprioceptive organs in the musculoskeletal system, it is unclear how such a system could integrate or predict visual or auditory consequences of an action without greatly increasing its complexity. One possibility, as proposed in a section of Grush (2004) on emulators in visual imagery, is that an emulator might make its predictions in an abstract amodal “spatial format” (i.e., a representation of the body and its surroundings in space), with sensory inputs being converted into this format for comparison and adjustment. Another possibility is that multiple emulators might operate in parallel for different modalities, allowing the motor system to compare its sensory predictions to different forms of input depending on the demands of a task. Regardless, as originally presented, the MET only allows for the simulation of unimodal (kinesthetic/proprioceptive) imagery.

The motor simulation theory, as usual, is difficult to categorize. Its core argument of functional equivalence between imagery and action seems to suggest that “imagery” constitutes the experience of generating and encoding a motor plan, which has no clear sensory consequences. However, Jeannerod (2001) also claims that the “covert” stage of action includes a representation of the actions' “consequences on the organism and the external world” and suggests elsewhere that forward modeling may play a role in motor imagery. Thus, whether MST is best categorized as unimodal or multimodal depends on the modalities one believes are involved in the forward modeling process.

### 3.4. How does motor imagery diverge from motor execution?

As motor imagery is generally thought to use similar neural pathways as movement, it is useful to compare how different theories believe that imagery and action diverge neurally. Generally, theories of imagery are categorized as being *functional equivalence* models, where imagery and action are thought to use the same neural pathways up to the point of execution, in contrast to *functional divergence* models that argue imagery requires pathways and cognitive systems beyond those used during action. However, this simple binary ignores a good deal of gray area between the two possibilities, making it perhaps better to think of functional equivalence as a spectrum based on the *degree* of neural overlap between imagery and action.

On one end of the functional equivalence spectrum are the motor simulation theory and motor emulation theory, which both explicitly argue that imagery uses the same neural motor pathways as action up to the point of actual movement, with the main point of divergence being that downstream motor commands to the muscles are inhibited during imagery through some process (either sub-threshold activation, active inhibitory neurons, or an increasing of the action threshold during imagery). There is growing evidence supporting the existence of such a process (e.g., Schwoebel et al., 2002; Grosprêtre et al., 2016), but its specific mechanism is still a matter of debate (Guillot et al., 2012; O'Shea and Moran, 2017). For the MET, another key difference is that imagery does not involve the updating of the emulator based on sensory input, given that there is no external input to compare with the internal image during mental simulation. Interestingly, despite functional equivalence being a core claim of MST, Jeannerod (2001) notes a number of differences in fMRI-measured neural activation between imagery and action and implies that differences in basal ganglia activation indicate cognitive differences between imagery and action. As such, the concept of functional equivalence may be better viewed as a guideline in these theories rather than a rule. The effects imagery model also falls near the functional equivalence end of the spectrum, as it hypothesizes an inhibition mechanism to prevent movement during imagery, argues that effects imagery is used equally during motor imagery and physical action, and suggests that the mechanism for deliberate motor imagery is the same associative mechanism that generates forward models during actual movement. However, the EIM is less aligned with functional equivalence than MST or MET, with Bach et al. (2021) suggesting that motor imagery requires more planning-related resources and less execution-related resources than overt action.

On the more functionally-divergent end of the spectrum is the motor-cognitive model, which argues that motor imagery requires cognitive resources beyond those used in motor execution in order to vividly simulate and monitor an imagined action. This theory implies that goal selection and high-level motor planning use the same neural pathways during motor imagery as in actual movement, but that the two diverge at the point of encoding: during movement, motor plans are encoded into muscle-specific motor format before being sent to the muscles. During imagery, the MCM argues that the initial image created by the high-level motor plan is then simulated in real-time and elaborated by drawing on attentional, executive, and memory resources to create a detailed image of the experience of that plan. Relative to MST and MET, the MCM implies that imagery practice should not affect the encoding of motor plans, and that motor action presumably uses a separate forward modeling process for learning during physical practice.

The perceptual-cognitive model falls somewhere between functional equivalence and functional divergence. Although its process of motor-cognitive reorganization is thought to occur during both physical and mental practice, imagery and action differ in the attention allocated to the reorganization process. During action, attention needs to be split between high-level motor planning and external sensory feedback, whereas during imagery it can be focused completely on the reorganization process. Frank and Schack (2017) argue that this can lead to diverging benefits of imagery and physical training, with physical practice resulting in better task performance but mental practice resulting in better and more expert-like cognitive representations of the movements involved in a complex action (as found by Frank et al., 2014). As such, a prediction that follows from the PCM is that biasing attention toward motor planning during physical practice should produce similar results to imagery: better cognitive representations of motor plans at the cost of worse motor plan encoding. It is important to note that the PCM makes no direct claims about the role of plan encoding in the imagery process: although its proposed mechanism for motor imagery does not *require* the mapping of motor plans to specific muscle commands, the PCM still allows that descending motor pathways may be activated as a result of activity in motor planning areas.

Neuroimaging evidence regarding functional equivalence is mixed. Although fMRI research supports that there are indeed many common areas of activation between imagery and execution of an action (e.g., pre-motor cortex, cerebellum, supplementary motor area), there are also some important differences (see O'Shea and Moran, 2017 for a review). Notably, activation of the primary motor cortex (M1) is weaker and less consistent during motor imagery than during actual movement, with two recent meta-analyzes reporting no consistent activation of M1 during motor imagery (Hétu et al., 2013; Hardwick et al., 2018). Additionally, within the areas active during both motor imagery and movement, there are often differences in the specific sub-regions involved (see Table 2 of O'Shea and Moran, 2017 for an overview). For example, although both motor imagery and action are associated with increased cerebellar activity, this activity is strongest in the posterior cerebellum during motor imagery whereas during movement it is strongest in the anterior cerebellum (O'Shea and Moran, 2017; Hardwick et al., 2018). Though these findings may seem to favor more functionally-divergent models of imagery, Hardwick et al. (2018) also report mixed evidence for the motor-cognitive model's predictions, with different methods of fMRI analysis yielding conflicting results as to whether motor imagery was associated with increased activity in the pre-frontal cortex relative to motor execution.

Evidence regarding functional equivalence from transcranial magnetic stimulation (TMS) studies has likewise been mixed. Favoring a more functionally-equivalent view, some research has found that TMS of the primary motor cortex has been shown to produce greater muscle responses when the participant is performing motor imagery with that muscle (Stinear et al., 2006), providing evidence that M1 is activated to some extent by motor imagery. Similarly, others have found that extensive prior imagery training can alter the directions of muscle responses to TMS stimulation of M1 (Yoxon and Welsh, 2019). Conversely, other studies have found that inhibiting M1 activity via TMS does not impair motor imagery performance (Kraeutner et al., 2017), whereas TMS inhibition of the dorsolateral pre-frontal cortex (dlPFC) has been shown to impair performance for motor imagery but not overt action (Martel and Glover, 2022), both consistent with a more functionally-divergent perspective.

## 4. Discussion and future directions

### 4.1. Combining mechanisms from different theories

Although there are many areas where different theories of motor imagery come into conflict (see Table 1 for a summary), it is perhaps more interesting to look at the areas where they do not. Specifically, although the exact processes involved in motor imagery differ between theories, there are a number of cases where theories' key mechanisms are not mutually exclusive and could thus be unified within a single model.

**Table 1:** A summary of the properties of different theories of motor imagery.

**Theory property**	**Motor simulation theory**	**Motor emulation theory**	**Motor-cognitive model**	**Perceptual-cognitive model**	**Effects imagery model**
Locus of experienced imagery	Ambiguous	Post-encoding	Pre-encoding	Pre-encoding	Pre-encoding
Primary learning mechanism	Long-term potentiation	Simulated feedback	Simulated feedback^*a*^	Cognitive reorganization	Long-term potentiation
Modalities involved	Kinaesthetic^*b*^	Kinaesthetic	Multimodal	Multimodal	Multimodal
Functional equivalence with action	Very high	Very high	Partial	High	High

^*a*^Although, Glover and Baran (2017) does not propose a learning mechanism for the motor-cognitive model (and explicitly note this as a limitation), its elaboration process offers a mechanism for simulated feedback.

^*b*^Because motor simulation theory implies that motor imagery's sensory results are the product of a forward model, the modalities involved in imagery depend on the modalities one believes are involved in forward modeling.

**Figure 7:**
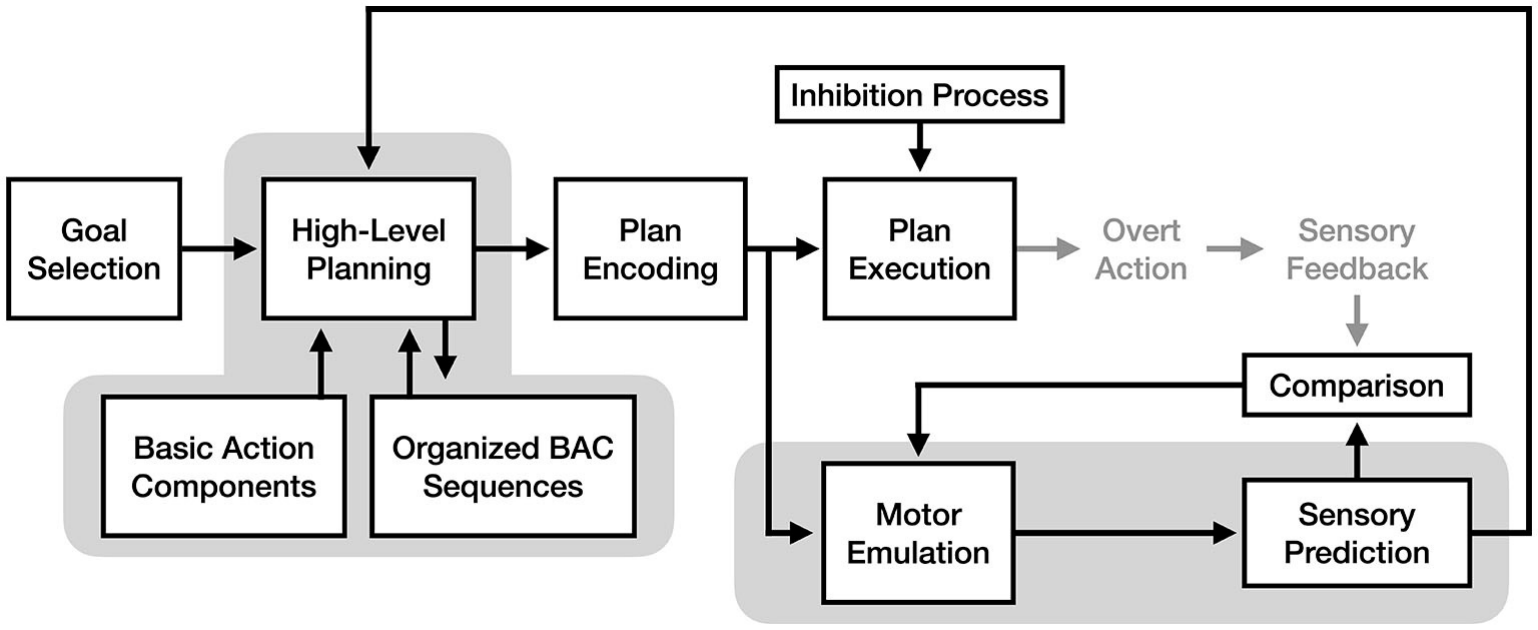
A fusion model of motor imagery combining the motor emulation theory's forward model mechanism with the perceptual-cognitive model's cognitive reorganization and chunking mechanism. The components of the model responsible for the experience of motor imagery are enclosed in gray.

One interesting fusion would be combining the motor emulation mechanism proposed by the motor emulation theory with the cognitive reorganization mechanism proposed by the perceptual-cognitive model. Because these mechanisms exist at different stages of the motor process, it is possible that *both* mechanisms are used as part of motor imagery, either in tandem or separately based on the demands of a given situation (see Figure 7). In an MET + PCM model, the emulation mechanism would be responsible for predicting how well a motor plan would accomplish a goal, while the reorganization process would optimize the future retrieval of that motor plan once it has been initially refined via simulation. This model would provide multiple complementary mechanisms for learning via motor imagery.

**Figure 8:**
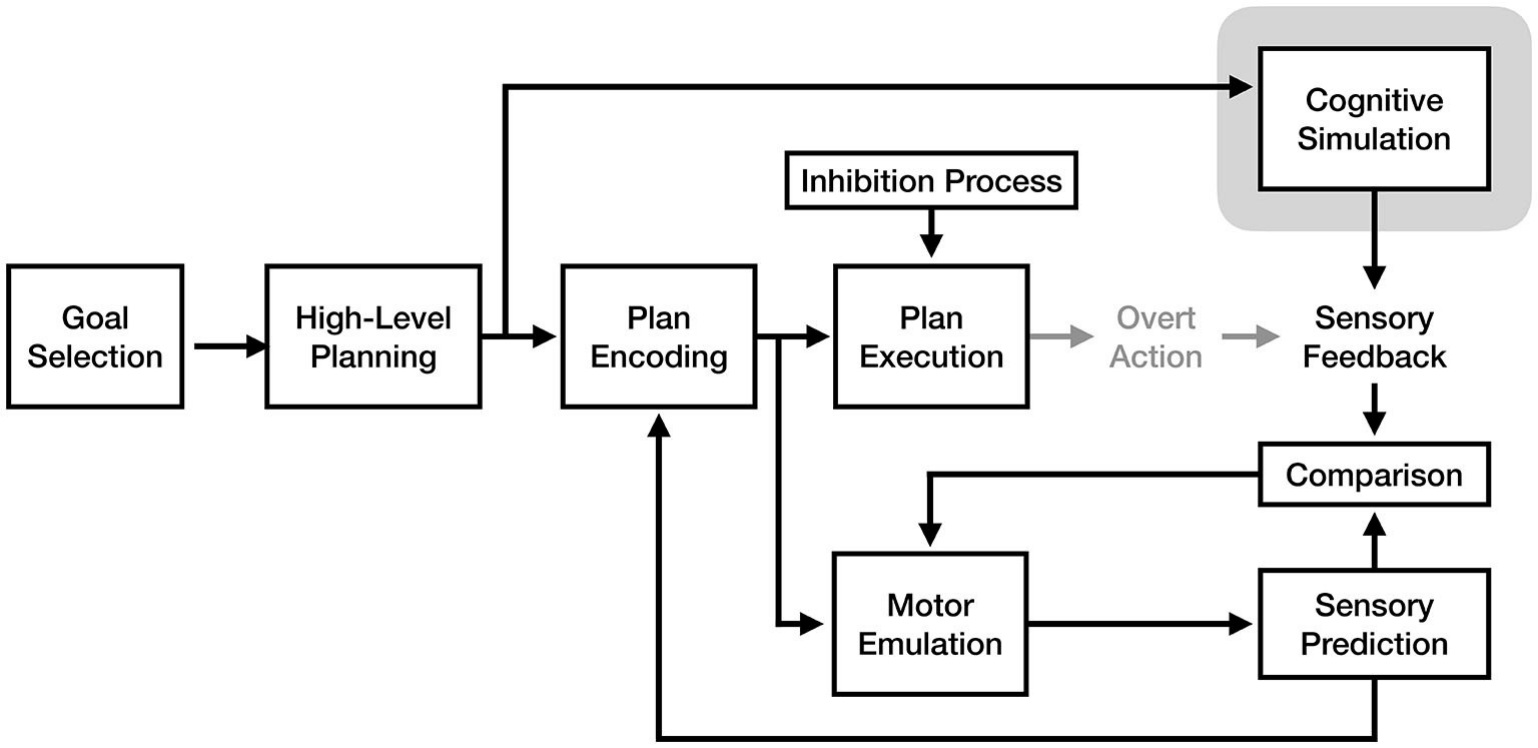
A fusion model of motor imagery combining the motor-cognitive model's cognitive simulation with the emulation and comparison mechanism from motor emulation theory. The component of the model responsible for the experience of motor imagery is enclosed in gray.

An additional potential fusion would be the vivid cognitive plan simulation of the motor-cognitive model with the sensory emulation and feedback mechanism of the motor emulation theory. Although both theories argue for different mechanisms for simulating motor plans, a possible combination of these mechanisms might be that both run in parallel: while the MET's emulator makes sensory predictions based on encoded motor plans, the MCM's active elaboration and monitoring process would simulate sensory input which could be compared with the emulator's predictions using its comparison mechanism (see Figure 8). This model is similar to the one proposed by Solomon et al. (2022), which likewise suggests that forward model predictions are compared with imagined movement during imagery.

Lastly, the concept of “effects imagery” (Bach et al., 2021) as an associative bridge between goal selection and motor plan initiation is compatible with all the theories of imagery under review. Given that the other models of motor imagery make no strong claims about how motor planning is initiated, the claim that conscious or subconscious imagery of desired effects initiates action does not conflict with any of the other mechanisms of effortful motor imagery suggested by the theories.

In general, the broad compatibility of different theories' mechanisms allows for some interesting mix-and-match combinations, but also raises a problem: if evidence for one mechanism of motor imagery isn't necessarily evidence against another, it becomes more difficult to determine which theory best fits the evidence. One solution might be to focus on testing specific mechanisms instead of entire theories, given their general modularity as illustrated above. If such research finds evidence for multiple mechanisms, this can be factored into future theory.

### 4.2. Does motor imagery require motor encoding?

To move our understanding of motor imagery forward, a key question highlighted by the theory comparison is whether motor imagery requires the encoding of motor plans into motoric format. If lesions or TMS inhibition of regions associated with encoding conscious plans into motor format reduce the effectiveness of motor imagery training, this would provide compelling evidence for an emulation mechanism as proposed by MET, as well as for the general functional equivalence of motor imagery and motor execution as proposed by MST. Conversely, evidence that disruption of plan encoding *does not* interfere with mental practice would strongly suggest that motor imagery's core mechanisms occur before plan encoding, as suggested by the PCM and MCM. If disruption of plan encoding *reduces* the benefits of mental practice without eliminating them completely, this would evidence the idea of multiple mechanisms involved in motor imagery, such as the PCM + MET fusion model proposed above. Existing work has examined how TMS inhibition of various motor-related brain regions impacts motor imagery performance (see Chepurova et al., 2022 for a review), yielding preliminary evidence that motor imagery is not impaired by disrupting encoding-related regions (specifically the primary motor cortex, Kraeutner et al., 2017). However, additional research with other relevant brain regions and a wider range of motor tasks is needed before any definitive conclusions can be drawn.

In addition to lesion or TMS disruption studies, another method of testing whether imagery requires plan encoding might be to test how motor imagery affects plan-to-effector mappings: if the encoding of high-level plans into motor format is rehearsed during motor imagery, the connections between a given plan and its associated motor commands would presumably be strengthened with repeated practice. Existing research on the lateralization of motor learning has found that actions rehearsed via motor imagery are less effector-dependent than those practiced physically, suggesting differences in the rehearsal of plan encoding between the two (Kraeutner et al., 2020). A potential paradigm to explore this further might be to have participants practice a directional motor task (e.g., pointing to on-screen targets with a joystick), have them perform imagery of the task, and then test them again on the task with the movement-outcome mappings of the original task inverted. If motor imagery involves plan encoding, participants who perform mental rehearsal of the initial “incorrect” mapping should take longer to adapt to the inverted mapping than those who performed no imagery at all.

Neuroimaging data may offer additional insight on the role of plan encoding but comes with important limitations. Firstly, given that neural structures are complex, interconnected, and typically involved in multiple functions, it is difficult to infer the meaning of an observed difference in activity without corresponding behavioral data. Additionally, the activation of an encoding-related region during imagery is not evidence that plan encoding is *necessary* for motor imagery, given that the activation could simply be unrelated downstream activation from motor planning processes. With those limitations in mind, a relevant finding from Hardwick et al. (2018) is that cerebellar lobule IV (thought to contain a neural map of the body) was consistently active during action but not motor imagery, implying indirectly that the encoding of movement plans to specific muscles may play less of a role during imagery than action. Additionally, as mentioned earlier, activation in the primary motor cortex (which is thought to be a key structure mapping motor plans to specific muscles) is generally weak or absent during motor imagery.

### 4.3. Integrating theory from similar fields

A notable limitation of most of the existing work on motor imagery is that it rarely draws upon theory or concepts from the related cognitive fields of non-motoric mental imagery and memory. Given that both these cognitive mechanisms are generally thought to play essential roles in motor learning and motor imagery, their respective bodies of research are likely to be useful in developing future models of motor imagery.

For example, the topic of non-motoric mental imagery (i.e., the internal simulation of sensory input) has generated a great deal of research over the past several decades, with such work offering detailed accounts about how such imagery is stored and retrieved (Kosslyn, 1981), the neural and cognitive mechanisms involved in imagery (Pearson et al., 2015), and its purpose in general cognition (Moulton and Kosslyn, 2009). Theory from this field, and from multimodal imagery research in particular (e.g., Lacey and Lawson, 2013), is likely to offer useful new insights and perspectives on the mechanisms involved in motor imagery.

Additionally, as motor learning involves the retrieval of previously-stored motor plans and sequences of actions, future theories of motor imagery may benefit from drawing on cognitive memory theory. As such retrieval and storage is presumably done through general cognitive mechanisms of long-term memory and working memory, a better understanding of the concepts and models in this field (particularly in the areas of procedural memory and skill learning, see Johnson, 2013 for a review) would help inform the design of future models of motor imagery, as well as ground them more clearly in the language used in broader cognitive science. Beyond the direct overlap of memory and motor imagery, the *conceptual* overlap between the fields (e.g., the study of the encoding, decoding, and rehearsal of patterns to improve performance) suggests that memory research is a rich potential source of concepts and paradigms that could be applied to similar questions in the study of motor learning and imagery.

### 4.4. Improving the clarity of theories

Regardless of its content, it is essential for any theory of motor imagery to be clear and understandable by its target audience. Because theory papers are often lengthy and discuss a wide range of background, concepts, and supporting evidence, it is important to make sure the theory's key testable claims can be easily identified and extracted by the reader. However, the only paper under review that does this is O'Shea and Moran (2017), who attempt to formally define motor simulation theory by distilling it into three short, testable postulates. Future work would benefit greatly from similar bullet-point summarization, ensuring readers (and the theorists themselves) clearly understand the core claims that define a given theory.

An additional suggestion to improve the clarity of future work is to include a diagram depicting how the proposed mechanism(s) of imagery relate to the motor system. The only theory reviewed that provides such an illustration is Grush (2004), which provides a detailed (albeit highly-technical) depiction that clearly conveys the mechanisms and flow of information hypothesized in motor emulation theory. Relatedly, future theory should make an effort to reuse the concepts and terminology of previous theory whenever possible, in order to facilitate comparison with previous work.

## 5. Conclusion

In this review, we summarize five current theories of motor imagery and identify their key similarities and differences. In addition, we highlight a number of future directions for models of motor imagery, including the merging of multiple theories and an increased focus on drawing from memory and non-motoric mental imagery research. We hope that these efforts make it easier for future researchers to relate their findings to current thinking in the field and help move theory forward.

## Author contributions

AH wrote the first draft of the manuscript and created the illustrations. Both authors gathered the bibliography for the review, contributed to manuscript revision, and approved the submitted version.

## Footnotes

^1^This was performed as an “expert” or “integrated” review and thus did not employ a systematic search as used in structured reviews (e.g., scoping reviews), as this was not the intent. The review was conducted by seeking out relevant theories and developing keywords based on those theories to find additional, relevant research.

^2^An review of current thinking on inhibitory mechanisms during motor imagery is beyond the scope of this work, but see Guillot et al. (2012), as well as Solomon et al. (2019) for a more recent discussion.

^3^Grush (2004) explicitly notes that MET uses the term “emulator” as a more descriptive synonym for “forward model,” clarifying that they refer to the same concept rather than separate, similar ones.
